# Pancreastatin Reduces Alternatively Activated Macrophages, Disrupts the Epithelial Homeostasis and Aggravates Colonic Inflammation. A Descriptive Analysis

**DOI:** 10.3390/biomedicines9020134

**Published:** 2021-02-01

**Authors:** Nour Eissa, Omar Elgazzar, Hayam Hussein, Geoffrey N. Hendy, Charles N. Bernstein, Jean-Eric Ghia

**Affiliations:** 1 Department of Immunology, University of Manitoba, Winnipeg, MB R3E 0T5, Canada or noureissa@live.com (N.E.); elgazzao@myumanitoba.ca (O.E.); 2 Children’s Hospital Research Institute of Manitoba, University of Manitoba, Winnipeg, MB R3E 3P4, Canada; 3 Section of Gastroenterology, Department of Internal Medicine, Rady Faculty of Health Sciences, University of Manitoba, Winnipeg, MB R3E 0T5, Canada; Charles.bernstein@umanitoba.ca; 4 The IBD Clinical and Research Centre, University of Manitoba, Winnipeg, MB R3A 1R9, Canada; 5 National Research Centre, Department of Parasitology and Animal Diseases, Veterinary Research Division, Giza 12622, Egypt; Hayam.Hussein.57@gmail.com; 6 Metabolic Disorders and Complications, McGill University Health Centre-Research Institute, Departments of Medicine, Physiology, and Human Genetics, McGill University, Montreal, QC H4A 3J1, Canada; geoffrey.hendy@mcgill.ca

**Keywords:** alternatively activated macrophages, chromogranin-A, gut hormones, intestinal epithelial cells, macrophages, mucosal drug action, pancreastatin, proinflammatory peptides

## Abstract

Ulcerative colitis (UC) is characterized by modifying alternatively activated macrophages (AAM) and epithelial homeostasis. Chromogranin-A (CHGA), released by enterochromaffin cells, is elevated in UC and is implicated in inflammation progression. CHGA can be cleaved into several derived peptides, including pancreastatin (PST), which is involved in proinflammatory mechanisms. Previously, we showed that the deletion of *Chga* decreased the onset and severity of colitis correlated with an increase in AAM and epithelial cells’ functions. Here, we investigated PST activity in colonic biopsies of participants with active UC and investigated PST treatment in dextran sulfate sodium (DSS)-induced colitis using *Chga^−/−^* mice, macrophages, and a human colonic epithelial cells line. We found that the colonic protein expression of PST correlated negatively with mRNA expression of AAM markers and tight junction (TJ) proteins and positively with mRNA expression of interleukin (IL)-8, IL18, and collagen in human. In a preclinical setting, intra-rectal administration of PST aggravated DSS-induced colitis by decreasing AAM’s functions, enhancing colonic collagen deposition and disrupting epithelial homeostasis in *Chga^+/+^* and *Chga^−/−^* mice. This effect was associated with a significant reduction in AAM markers, increased colonic IL-18 release, and decreased TJ proteins’ gene expression. In vitro, PST reduced *Chga^+/+^* and *Chga^−/−^* AAM polarization and decreased anti-inflammatory mediators’ production. Conditioned medium harvested from PST-treated *Chga^+/+^* and *Chga^−/−^* AAM reduced Caco-2 cell migration, viability, proliferation, and mRNA levels of TJ proteins and increased oxidative stress-induced apoptosis and proinflammatory cytokines release. In conclusion, PST is a CHGA proinflammatory peptide that enhances the severity of colitis and the inflammatory process via decreasing AAM functions and disrupting epithelial homeostasis.

## 1. Introduction

Alteration of the homeostatic balance in the gastrointestinal tract (GIT) has been implicated in the development of inflammatory bowel disease (IBD) that includes Crohn’s disease (CD) or ulcerative colitis (UC) [1]. It is considered that IBD results from an inappropriate response to luminal contents such as microbiota, dietary antigens and a breakdown of the intestinal barrier associated with an imbalance of the mucosal immune system [1]. In the mucosal immune system, macrophages represent the largest mononuclear phagocytes population and play a significant role in maintaining the GIT homeostasis [2]. Macrophages can be mainly subdivided into two major subtypes: proinflammatory macrophages and alternatively activated macrophages (AAM) [3,4]. At the difference of proinflammatory macrophages implicated in acute inflammation development, AAM produces large amounts of interleukin (IL)-10, scavenger- and mannose-receptors and are involved in terminating the inflammatory immune response and in regulating wound repair [5]. Mice portraying a deficient macrophage polarization towards the AAM phenotype exhibit a higher susceptibility to colitis [6]. Therefore, identifying a mediator that increases the polarization toward AAM or blocking one that can decrease it and its subsequent specific effects on the intestinal epithelial cells (IECs) and the intestinal barrier function would provide a better understanding to control and manage colonic inflammation.

The IECs form a physical and biochemical barrier to commensal and pathogenic microorganisms. They can sense and respond to stimuli to support their barrier function and contribute to the synchronization of appropriate immune responses [7,8]. Thus, IECs maintain an essential immunoregulatory function to sustain a robust intestinal barrier. As an example, lipopolysaccharide (LPS) plays a crucial pathogenic role in intestinal inflammation and a fundamental role in inducing intestinal injury by disturbing the tight junction (TJ) barrier [9,10,11]. Therefore, the functional dysregulation of IECs is an integral part of the intestinal defective intestinal TJ barrier that is a critical etiological factor leading to subsequent development of inflammatory response [9,10]. A loss of intestinal barrier function contributes to the systemic immune activation seen in IBD [7]. During intestinal inflammation, macrophage infiltration to the submucosa close to the epithelial layer suggests the existence of a crosstalk between these two cells [12]. Thereby, macrophages can play a significant role in the regulation of epithelial functions. Hence, a comprehensive understanding of the crosstalk between IECs and macrophages could help develop new strategies to prevent and treat IBD.

In addition, alteration of the endocrine system is implicated in IBD’s pathophysiology and is linked to a change in enterochromaffin cells number and related content: chromogranin-A (CHGA) [13,14]. Previously, we demonstrated that the deletion of *Chga* reduced the onset and severity of intestinal inflammation associated with an increase in AAM activity [11,15]. CHGA can be cleaved into several CHGA-derived peptides (CgDPs), notably catestatin (CST) and chromofungin [16,17]. Surprisingly, administration of CST or chromofungin decreases the clinical severity of experimental colitis and reduces the production of the proinflammatory cytokines by macrophages [18,19,20,21]. This unexpected result underscores the possible action of a dominant CgDPs with pro-inflammatory features. PST has recently been described to exert dysglycemic effects and to promote adipose tissue inflammation [22].

Taken together, since *Chga*-knockout mice showed a reduction in the preclinical setting of colitis [23], and some CgDPs protected against colitis [4,10,11,20,24,25], we hypothesized that PST could be one of the proinflammatory CgDPs that enhance the colonic inflammatory process through an alteration of the functional capacities of AAM and subsequently IECs. This study quantified and correlated PST’s level with different AAM and epithelial barrier markers in human colonic biopsies collected from persons with endoscopically active UC and using the dextran sodium sulfate (DSS) experimental model of colitis. Using cell culture models, we evaluated the effects of PST on the functions of AAM and IECs. Our translational study indicates that PST plays a crucial role in developing colonic inflammation in participants with UC and experimentally.

## 2. Material and Methods

### 2.1. Active UC Participants and Healthy Individuals

The human study was approved by the University of Manitoba Health Research Ethics Board (Project ID: HS14878 [E], Approval date: 17 January 2015). Participants with ulcerative colitis and healthy controls were recruited from the University of Manitoba IBD Clinical and Research Center. Endoscopic biopsies were obtained from the inflamed colonic mucosa of ten participants with active UC and non-inflamed tissues of ten healthy individuals. Healthy controls did not have any history of UC and were not under medication or chronic inflammatory conditions. Participants with active UC were not receiving immunosuppressive therapy. The individuals were between 27 and 55 years and with a mean age of 40 years. Participants gave their informed consent before the beginning of the study.

### 2.2. Animals

The University of Manitoba Animal Ethics Committee approved this study under Canadian animal research guidelines (Project ID: 15-010, Approval date: 23 March 2015). Mice heterozygous for *Chga* expression (*Chga^+/−^*) on a C57BL/6 background were utilized to generate *Chga^+/+^* and *Chga^−/−^* mice. Six to eight-week-old male *Chga^+/+^* and *Chga^−/−^* mice (23–25 g) were used. Mice were maintained in the animal care facility at the University of Manitoba under a specific pathogen-free barrier. Animals were kept in a 12-h dark/light cycle and fed ad libitum.

### 2.3. Peptides

Human PST (CHGA_273–301_: PEGKGEQEHSQQKEEEEEMAVVPQGLFRG-amide) and PST scrambled peptide (sPST: PEKGEEQLGEQVSEEGRQMAGVPHKQEEF) were synthesized by the solid-phase method and purified by reverse-phase high-performance liquid chromatography to <98% purity (Pepmic Co., Suzhou, China). The effective dose of 2.5 mg/kg/day, as reflected by previously published data related to peptide use, was administrated via intra-rectal (i.r.) administration [20,26]. Control experimental groups received phosphate buffer saline (1% PBS, i.r.). The scrambled peptide (sPST) was injected as a positive internal control to demonstrate PST’s sequence specificity.

### 2.4. In Vivo Acute Dextran Sulfate Sodium (DSS)-Induced Colitis

Dextran sodium sulfate (DSS) colitis was induced in mice by administering DSS 5% (wt/vol) for five days in the drinking water (molecular weight [MW], 40 kDa: MP Biomedicals, Soho, OH, USA) [27] to 6–8-week-old male *Chga^+/+^* and *Chga^−/−^* mice. Controls were time-matched and consisted of mice that received regular drinking water. The mean of DSS consumption was noted per cage daily.

### 2.5. External Disease Activity Index and Microscopic Assessment of Colitis

To assess the onset and severity of colitis, weight loss, stool consistency, and bleeding were reported [28,29,30] from day zero to day five during DSS treatment. Blood in the stool was assessed using the Hemoccult II test (Beckman Coulter, Oakville, ON, Canada). Mice were sacrificed on day five. Collagen deposition and fibrosis scores were assessed as described previously [31]. Colonic sections isolated from the splenic flexure were formalin-fixed (sigma, Mississauga, ON, Canada), paraffin-embedded (sigma), cut into 3 mm sections, and stained using Masson’s trichrome (sigma). Collagen deposition and fibrosis were scored based on a published scoring system that considers collagen deposition (score 0 = no increase, score 1 = increase in the submucosa, 2 = increase in the mucosa, 3 = increase in the muscularis mucosa and its thickening, 4 = increase in the muscularis propria and 5 = gross disorganization in the muscularis propria) and the percent involvement (score 1 = 1–25%, score 2 = 26–50%, score 3 = 51–75% and score 4 = 76–100%) [32].

### 2.6. Macrophage Cell Culture

#### 2.6.1. Macrophages Isolation from Colitic PST, sPST, and Non-PST Treated Groups

Five days after the beginning of the DSS treatment, resident peritoneal macrophages were isolated from all groups as described by Mosser and Zhang [33] and us [3]. Isolated macrophages were cultured in 2 mL Dulbecco’s modified Eagle’s medium (DMEM) supplemented with 100 unit/mL penicillin, 100 μg/mL streptomycin, and 10% deactivated fetal bovine serum (FBS). Cell cultures were incubated in a humidified 5% CO2 incubator at 37 °C. The overall cell viability of the adherent cell was greater than 95%.

#### 2.6.2. Macrophage Isolation from Naïve Mice

Peritoneal macrophages were isolated from naïve 6–8-week-old male *Chga^+/+^* and *Chga^−/−^* mice, then serum-starved overnight in DMEM with low FBS (0.5%). Macrophages were washed three times with 1% PBS solution and pretreated with PST (200 ng/mL) for four hours and then exposed for an additional 6 h to 1% PBS in medium or IL-4/IL-13 (20 ng/mL) [33] to induce AAM. After six hours, cells and supernatant medium were harvested for analysis.

### 2.7. Human Intestinal Epithelial Cell Line

A human intestinal epithelial cell line, Caco-2 (ATCC, Manassas, VA, USA), was cultured as we described previously [9]. For each experimental setup, three separate experiments were performed, and at least six wells per condition were assigned.

#### 2.7.1. Lipopolysaccharides (LPS)- and DSS- Stimulated Epithelial Cells in the Presence or Absence of PST-Treated Polarized AAM Supernatants

Two mL of AAM supernatant or naïve PBS-treated macrophage supernatant was added to the Caco-2 cell line for 24 h. Then, Caco-2 cells were challenged with LPS (1 μg/mL) (Escherichia coli serotype 127: B8, Sigma-Aldrich, St. Louis, MO, USA) or 5% DSS for an additional 24 h [34]. TJ proteins mRNA levels, proinflammatory cytokines IL-8 and IL-18, migration, proliferation, viability and oxidative stress survivability of IECs were evaluated.

#### 2.7.2. Epithelial Cell Migration Assessed Using a Wound-Healing Assay

Two milliliters of AAM supernatant or naïve PBS-treated macrophage supernatant was added to the Caco-2 cell line for 24 h. Caco-2 cells were wounded using a sterile 100-μL pipette tip dragged perpendicular to a black line drawn on the plate’s underside for reference. Images were taken at wounding (0) and 48 h later using an Evos FL imaging system at 4× magnification. Wound widths were determined by averaging six measurements per image. Only scratches with edges captured in one frame at the time point 0 h were included for final analysis. Measurements were taken from edge-to-edge at the time point 0 h and compared with measurements at the 48 h time point using ImageJ (National Institutes of Health, Bethesda, MD, USA) software [35]. Reported values considered the difference between the 0 and 48 h time points, with higher values representing increased cellular migration.

#### 2.7.3. Epithelial Proliferation and Viability Assessed Using Cell Numbers and MTT Assay

Intestinal epithelial viability was studied in vitro using the 3-(4, 5-dimethyl thiazolyl-2yl)-2, 5-diphenyl tetrazolium (MTT) assay. Two mL of AAM supernatant or naïve PBS-treated macrophage supernatant was added to the Caco-2 cell line for 24 h. After 72 h, the media aspirated and cells quantified by MTT assay (Trevigen Inc, Gaithersburg, MD, USA) according to the manufacturer’s instructions. The plates were quantified using a microplate spectrophotometer (Molecular Devices, Sunnyvale, CA, USA) at a wavelength of 570 nm.

#### 2.7.4. Epithelial Cell Survival Using an Oxidative Stress Assay

Two milliliters of AAM supernatant or naïve PBS-treated macrophage supernatant was added to the Caco-2 cell line for 24 h, and two mL of 200 mmol/L of H2O2 in phosphate-buffered saline (PBS) was added for 30 min. Trypan blue staining was used to count viable cells.

### 2.8. Quantitative Real-Time Reverse-Transcription Polymerase Chain Reaction

Total RNA was extracted using TRIzol™ Plus RNA purification kit (Life Technologies, NY, USA) and reverse-transcribed using SuperScript VILO cDNA Synthesis Master Mix (Invitrogen, Grand Island, NY, USA) following the manufacturer instructions. A quantitative polymerase chain reaction (RT–qPCR) was used to quantify gene expression in a Roche light cycler 96 Real-Time system using power SYBR green master mix (Life Technologies, Burlington, ON, USA). Difference in the threshold cycle (ΔCt) number between the target genes and mouse eukaryotic elongation factor 2 (*Eef2*) and human TATA box binding protein (TBP) as optimal reference genes [26,36,37] were used to calculate differences in the transcript expression using ΔCt method. Human and mice primers sequences for cytokine, AAM, genes of TJ proteins and IECs markers provided in Table 1 and Table 2.

### 2.9. Enzyme-Linked Immunosorbent Assay (ELISA)

Cytokines released and arginase activity measurements were performed on clarified full-thickness colon homogenates from mice and supernatants collected from the peritoneal macrophages cell culture using ELISA. Colonic samples were homogenized mechanically in 700 mL of Tris-HCl buffer containing protease inhibitors (sigma, Mississauga, ON, Canada), then centrifuged for 30 min, and supernatants were frozen at 80 °C until assay [20]. Commercial ELISA kits for mouse IL-10, mouse IL-18, human IL-8 and human IL-18 (R&D Systems, Inc., Minneapolis, MN, USA), and mouse arginase activity (Abnova, Walnut, CA, USA) were used.

### 2.10. Data Analysis

Unpaired a Mann–Whitney U test, and one- and two-way ANOVA followed by a post hoc test when appropriate were used to compare the different groups. Spearman’s correlation test was used. *P* values (two-tailed) below 0.05 considered significant. Data are presented as the mean ± standard error of the mean (sEM). Statistics computed using GraphPad Prism software (version 6; GraphPad Software, Inc, La Jolla, CA, USA).

## 3. Results

### 3.1. PST Is Increased in Participants with Active UC and Correlates with mRNA Expression of AAM, TJ Proteins, Epithelial Cells Associated Cytokines and Collagen in Human

**Figure 1:**
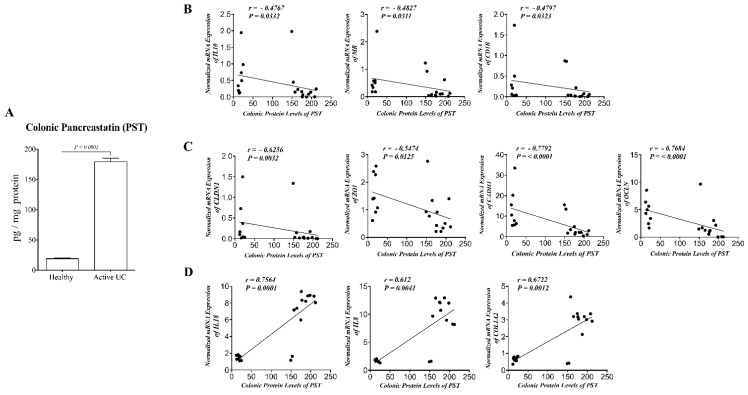
Pancreastatin (PST) is increased in participants with active ulcerative colitis (UC) and correlates negatively with mRNA expression of alternative-activated macrophages (AAM) markers and tight junction (TJ) protein, and correlates positively with epithelial cells-associated cytokines and collagen expression in human. (**A**) Protein level of PST in UC participants’ colonic tissue (*n* = 10) and healthy individuals (*n* = 10). Correlation analysis between PST protein level and mRNA expression of (**B**) AAM markers (*IL10*, mannose receptor *[MR]*, Cluster of differentiation 1B *[CD1B]*), (**C**) TJ proteins (claudin *[CLDN1]*, zonula occludens-1 *[ZO1]*, E-cadherin *[CDH1]* and occludin *[OCLN])*, (**D**) and *IL8*, *IL18*, and collagen (*COL1A2*). ELISA quantified protein level. Quantitative real-time RT–PCR quantified mRNA expression. The relative mRNA expression was determined by calculating the difference in the threshold cycle (ΔCt) method. Mann–Whitney test and Spearman’s correlation method were used to analyze the data. Two tails significance level adjusted at 0.05.

PST’s biopsy protein level demonstrates an 8.98-fold increase in participants with active UC (*n* = 10) compared with healthy control (*n* = 10) (Figure 1A). Furthermore, biopsy’s protein level of PST correlated negatively with mRNA expression of AAM markers (*IL10*, mannose receptor (*MR*), *CD1B*) (Figure 1B) and mRNA expression of TJ protein markers (*CLDN1, ZO1, CADH1, OCLN*) (Figure 1C) in human. However, PST showed a strong positive relationship with mRNA expression of epithelial-associated cytokines (*IL8, IL18*) and collagen (*COL12A*) (Figure 1D) in humans. Moreover, mRNA expression of PST (*CHGA Exon-VII*) was significantly increased, showing a 41-fold increase in the colonic biopsies from participants with active UC compared with healthy control (Figure S1A). Furthermore, mRNA expression of PST (*CHGA Exon-VII*) correlated negatively with mRNA expression of AAM markers (Figure S1B) and mRNA expression of TJ protein (Figure S1C) in humans. PST demonstrated a strong positive relationship with mRNA expression of epithelial-associated cytokines and collagen (Figure S1D) in humans.

### 3.2. PST Is Increased, and Treatment Aggravates the Disease Activity in DSS-Induced Colitis

**Figure 2:**
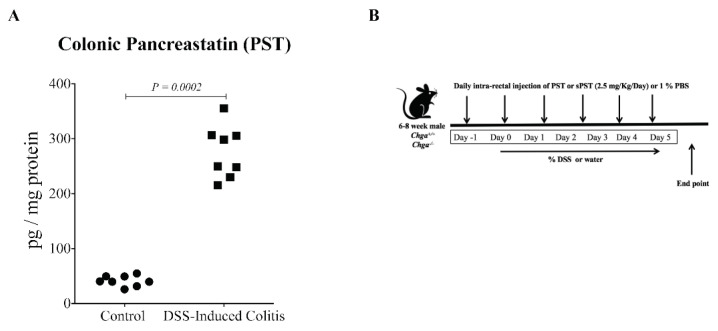

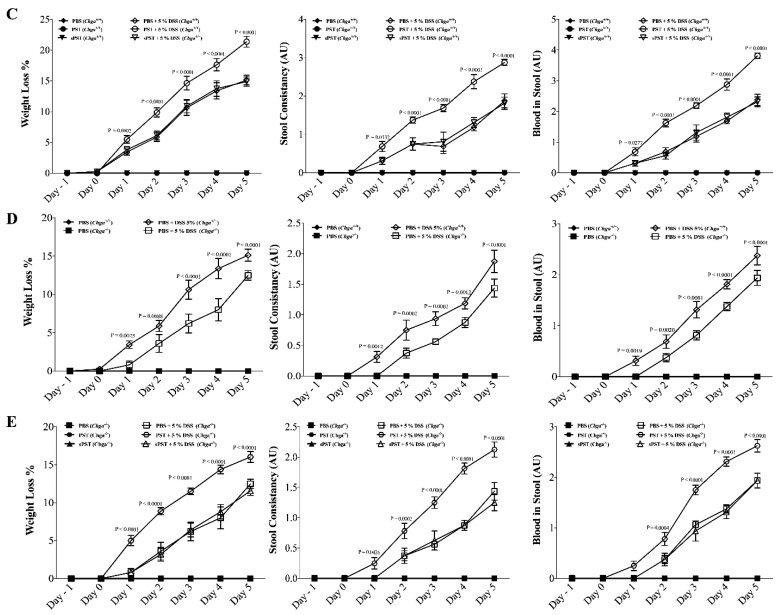
Pancreastatin (PST) aggravates the onset and severity of dextran sulfate sodium (DSS)-induced colitis. C57BL/6 background wild-type (*Chga*^+/+^) and CHGA-knockout mice (*Chga^−/−^*) were given 5% DSS solution in their drinking water for five days to induce colitis. Control mice received water without DSS. PST or scrambled peptide (sPST) (2.5 mg/kg/day, intrarectal) and 1% phosphate buffer saline (1% PBS) treatment started one-day before induction of colitis. (**A**) Colonic protein expression of PST in colitic *Chga^+/+^* mice. (**B**) Illustration depicting the experimental design. (**C**) Weight loss percentage, stool consistency, and blood in the stool of colitic *Chga^+/+^* treated with PST, sPST or 1% PBS. (**D**) Weight-loss percentage, stool consistency, and blood in the stool of colitic *Chga^−/−^* (Previously published [11]). (**E**) Weight-loss percentage, stool consistency, and blood in the stool of colitic *Chga^−/−^* treated with PST, sPST or 1% PBS. AU = arbitrary unit. Values are shown as the mean ± SEM, *n* = 8–10 mice/group and the *p*-value was adjusted at 0.05. Mann–Whitney U test was used to compare the PST level in non-colitic and colitic *Chga^+/+^* mice. Two-way ANOVA, followed by multiple comparison tests, was applied to compare the markers in the different groups.

We found that PST’s colonic expression was significantly upregulated during colitis in wild-type mice (*Chga^+/+^*) compared with control (Figure 2A). Next, we investigated the consequences of PST exogenous administration during DSS-induced colitis (Figure 2B). We found that PST treatment increased the onset of the severity of DSS-induced colitis in *Chga^+/+^* mice represented by an increase in weight loss, stool consistency and blood in the stool compared with colitic PBS-treated *Chga^+/+^* mice (Figure 2C). As previously described and published [4,11], the deletion of CHGA reduced the onset and progression of intestinal inflammation in DSS-treated *Chga^−/−^* mice compared to DSS-treated *Chga^+/+^* mice (Figure 2D). However, PST treatment aggravated DSS-induced colitis development in *Chga^−/−^* mice compared with PBS-treated colitic *Chga^−/−^* mice (Figure 2E). Furthermore, sPST did not significantly affect the markers in *Chga^+/+^* and *Chga^−/−^* mice (Figure 2C,E).

### 3.3. PST Treatment Promotes Colonic Expression and Deposition of Collagen in DSS-Induced Colitis

**Figure 3:**
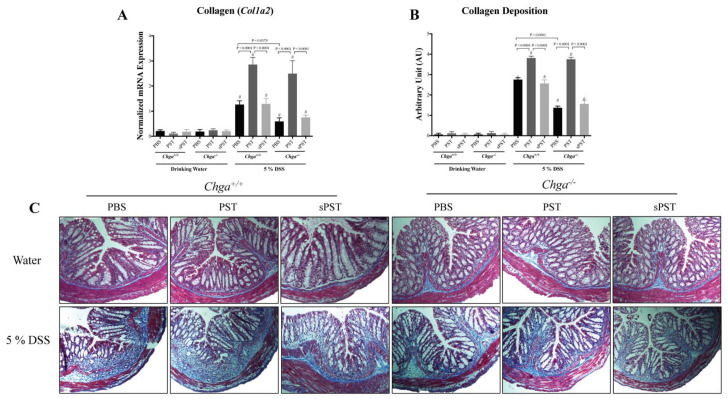
Pancreastatin (PST) promotes collagen expression and deposition in the colon during dextran sulfate sodium (DSS)-induced colitis. C57BL/6 background wild-type (*Chga^+/+^*) and CHGA-knockout mice (*Chga^−/−^*) were given 5% DSS solution in their drinking water for five days to induce colitis. Control mice received water without DSS. PST or scrambled peptide (sPST) (2.5 mg/kg/day, intrarectal) and 1% phosphate buffer saline (1% PBS) treatment started one-day before induction of colitis. Colonic collagen deposition scores were quantified by (**A**) quantitative real-time RT–PCR in mRNA expression of collagen col1a2 or (**B**,**C**) Masson trichrome (100×), whereas collagen was stained in blue. The relative mRNA expression was determined by calculating the difference in the threshold cycle (ΔCt) method. One-way ANOVA followed by multiple comparison tests. Each value represents the mean ± SEM, *n* = 8–10 mice/group. # refers to significance compared with control groups (drinking water).

Colitic PST-treated *Chga^+/+^* mice showed a significant upregulation in the colonic mRNA expression of collagen (*Col1a2*) (Figure 3A) and collagen deposition score (Figure 3B). This was associated with increased tissue architecture’s loss, edema, and mixed immune cell infiltrate (mononuclear cells, neutrophils, and eosinophils) when compared with colitic PBS-treated *Chga^+/+^* mice (Figure 3C). While *Chga^−/−^* mice exhibited a decrease in colonic mRNA expression of *Col1a2*, collagen deposition score and colonic damage (Figure 3A–C, PST administration reverted these effects (Figure 3A–C to the level of colitic PST-treated *Chga^+/+^* mice (Figure 3A–C. Furthermore, sPST did not significantly affect the onset and severity of DSS-induced colitis in *Chga^+/+^* and *Chga^−/−^* mice (Figure 3).

### 3.4. PST Treatment Promotes IL-18 Colonic Expression and Alters TJ Proteins Colonic Expression in DSS-Induced Colitis

**Figure 4:**
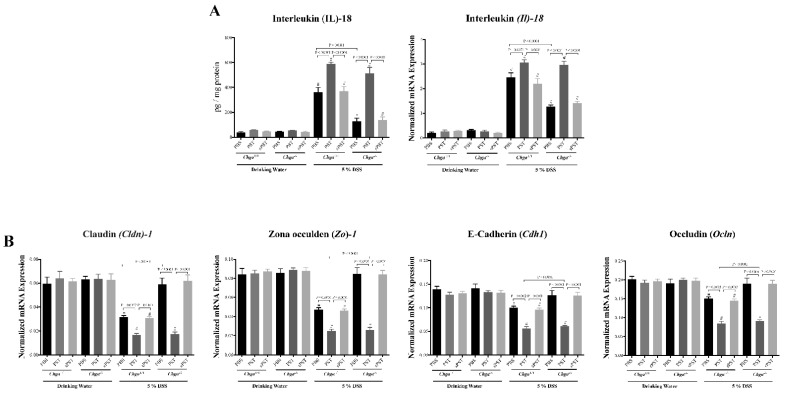
Pancreastatin (PST) increases IL-18 release and alters mRNA expression of tight junction (TJ) protein in dextran sulfate sodium (DSS)-induced colitis. C57BL/6 background wild-type (*Chga^+/+^*) and CHGA-knockout mice (*Chga^−/−^*) were given 5% DSS solution in their drinking water for five days to induce colitis. Control mice received water without DSS. PST or scrambled peptide (sPST) (2.5 mg/kg/day, intrarectal) and 1% phosphate buffer saline (1% PBS) treatment started one-day before induction of colitis. (**A**) Colonic protein level and mRNA expression of *Il-18*. (**B**) Colonic mRNA expression of TJ protein claudin (*Cldn1*), zonula occludens-1 (*Zo1*), E-cadherin (*Cdh1*) and occludin (*Ocln*). ELISA quantified protein level. Quantitative real-time RT–PCR quantified mRNA expression. The relative mRNA expression was determined by calculating the difference in the threshold cycle (ΔCt) method. One-way ANOVA followed by multiple comparison tests. Each value represents the mean ± SEM, *n* = 8–10 mice/group. # refers to significance compared with control groups (drinking water).

IECs homeostasis is maintained by TJ proteins and IL-18, which are critical aspects of intestinal inflammation [38,39]. Colitic PST-treated *Chga^+/+^* mice revealed a significant increase in the colonic protein levels and mRNA expression of IL-18 (Figure 4A) associated with a significant decrease in the colonic mRNA expression of TJ markers (*Cldn1*, *Zo1*, *Cdh1*, *Ocln*) (Figure 4B) when compared with colitic PBS-treated *Chga^+/+^* mice. Colitic PBS-treated *Chga^−/−^* mice exhibited a significant decrease in IL-18, but no significant modification of TJ proteins’ mRNA expression was demonstrated with non-colitic PBS-treated *Chga^+/+^* mice (Figure 4A,B). However, in colitic *Chga^−/−^* mice, PST treatment reversed IL-18 colonic level and mRNA expression of TJ markers (Figure 4A,B). Administration of the sPST peptide did not modify the markers studied.

### 3.5. PST Treatment Decreases Colonic AAM Markers and Decreases AAM-Associated Anti-Inflammatory Mediators in DSS-Induced Colitis

**Figure 5:**
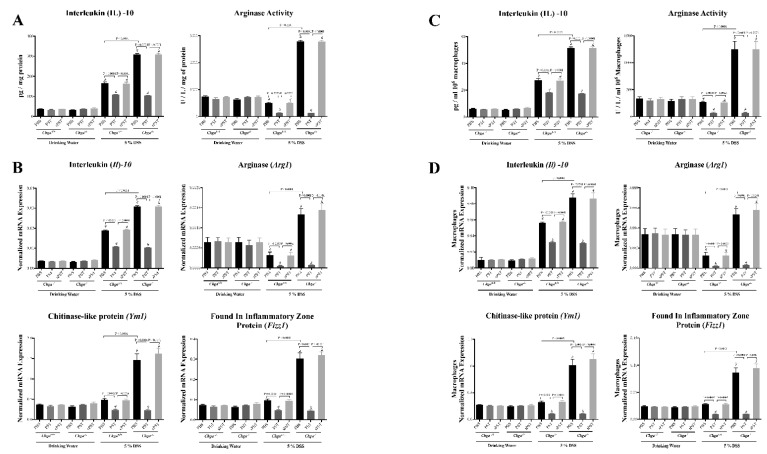
Pancreastatin (PST) treatment decreases colonic and peritoneal isolated macrophages AAM markers and AAM-associated anti-inflammatory mediators in dextran sulfate sodium (DSS)-induced colitis. C57BL/6 background wild-type (*Chga^+/+^*) and CHGA-knockout mice (*Chga^−/−^*) were given 5% DSS solution in their drinking water for five days to induce colitis. Control mice received water without DSS. PST or scrambled peptide (sPST) (2.5 mg/kg/day, intrarectal) and 1% phosphate buffer saline (1% PBS) treatment started one-day before induction of colitis. (**A**) Colonic protein levels of interleukin (IL)-10 and arginase activity and (**B**) colonic mRNA expression of AAM markers (*Il10*, arginase [*Arg1*], Chitinase-like protein [*Ym1*], *and* Found in inflammatory zone protein [*Fizz1*]). (**C**) Protein levels of IL-10, arginase activity, and (**D**) mRNA expression of AAM markers (*Il10, arg1, Fizz1, and Ym1*) in peritoneal macrophages isolated from the different groups. ELISA quantified protein level and activity. Quantitative real-time RT–PCR quantified mRNA expression. The relative mRNA expression was determined by calculating the difference in the threshold cycle (ΔCt) method. One-way ANOVA followed by multiple comparison tests. Each value represents the mean ± SEM, *n* = 8–10 mice/group. # refers to significance compared with control groups.

AAM produces IL-10, arginase and many extracellular molecules that play a crucial role in intestinal homeostasis and tissue repair [40,41]. Therefore, to further determine PST’s role in modifying antigens presenting cell markers during colitis progression, AAM and their mediators were explored. Colitic PST-treated *Chga^+/+^* mice displayed a significant decrease in colonic IL-10 level and arginase activity (Figure 5A) associated with a downregulation in colonic mRNA expression of *Il10*, *Arg1*, *Ym1*, and *Fizz1* when compared with colitic PBS-treated *Chga^+/+^* mice (Figure 5B). Conversely, colitic PBS-treated *Chga^−/−^* mice exhibited a significantly increased level of colonic IL-10 and arginase activity associated with an upregulation of mRNA expression of AAM markers (*Il10*, *Arg1*, *Ym1*, *Fizz1*) when compared with colitic PBS-treated *Chga^+/+^* mice (Figure 5A,B). In colitic *Chga^−/−^* mice, PST treatment decreased these markers (Figure 5A,B).

To determine the cell implicated in this mechanism, we next probed the role of PST on macrophage function.

Peritoneal macrophages isolated from colitic PST-treated *Chga^+/+^* mice displayed a significant decrease in colonic IL-10 level and arginase activity (Figure 5C) associated with a significant downregulation in colonic mRNA expression of *Il10*, *Arg1*, *Ym1*, and *Fizz1* when compared with colitic PBS-treated *Chga^+/+^* mice (Figure 5D). Conversely, peritoneal macrophages isolated from colitic PBS-treated *Chga^−/−^* mice exhibited a significantly increased level of colonic IL-10 and arginase activity associated with an upregulation in mRNA expression of AMM markers (*Il10*, *Arg1*, *Ym1*, *Fizz1*) when compared with colitic PBS-treated *Chga^+/+^* mice (Figure 5C,D). In colitic *Chga^−/−^* mice, PST treatment decreased these markers (Figure 5C,D).

In both experimental plans, colonic mucosa or isolated peritoneal macrophages, sPST peptide administration did not modify AAM markers.

### 3.6. In Vitro, PST Treatment Decreases the Functional Capacity of AAM

**Figure 6:**
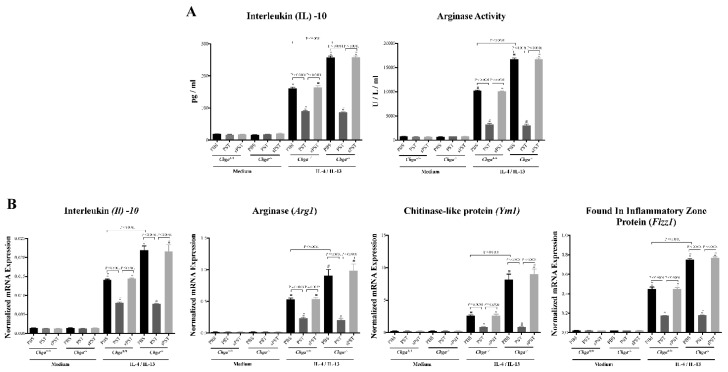
In vitro, pancreastatin (PST) pretreatment decreases the functional capacity of naïve macrophages polarized toward an alternative-activated macrophages profile (AAM). Peritoneal macrophages were isolated from naïve *Chga^+/+^* and *Chga^−/−^* mice. Macrophages were pretreated with PST or sPST (200 ng/mL) or 1% PBS for four hours, then stimulated by interleukin (IL)-4/IL-13 (20 ng/mL) for six h and conditioned medium was harvested. (**A**) Medium protein levels of IL-10 and arginase activity. (**B**) Cell mRNA expression of AAM markers (*Il10*, arginase [*Arg1*], chitinase-like protein [*Ym1*], and Found in inflammatory zone protein [*Fizz1*]). ELISA quantified protein level and activity. Quantitative real-time RT–PCR quantified mRNA expression. The relative mRNA expression was determined by calculating the difference in the threshold cycle (ΔCt) method. One-way ANOVA followed by multiple comparison tests. Each value represents the mean ± SEM, *n* = 9 mice/group. # refers to significance compared with medium control groups. Each experiment was repeated at least three times.

Previously, we demonstrated that CHGA and its derived peptides modulate macrophages’ functional activity during colitis progression [15,18,19,20,42,43]; therefore, herein, we determined if PST can directly modulate AAM’s polarization. Peritoneal macrophages of naïve *Chga^−/−^* and *Chga^+/+^* mice were isolated and pretreated with PST, then polarized toward an AAM profile using IL-4/IL-13. PST-pretreatment of *Chga^+/+^* macrophages polarized to an AAM profile displayed a significant decrease in IL-10 release and arginase activity (Figure 6A) and was associated with a significant downregulation in mRNA expression of *Il10*, *Arg1*, *Ym1*, and *Fizz1* when compared with PBS-pretreated *Chga^+/+^* macrophages polarized to an AAM profile (Figure 6B). However, PBS-pretreatment of *Chga^−/−^* macrophages polarized to an AAM profile exhibited a significant increase in IL-10 release and arginase activity and was associated with significant upregulation in mRNA expression of *Il10*, *Arg1*, *Ym1*, *Fizz1* when compared with PBS-treated *Chga^+/+^* macrophages polarized to an AAM profile (Figure 6A,B). Remarkably, PST pretreatment of *Chga^−/−^* macrophages polarized to an AAM profile decreased these markers (Figure 6A,B). Administration of the sPST peptide did not modify the markers studied.

### 3.7. Conditioned Medium from PST-Pretreated Macrophages Polarized to an AAM Profile Increases IL-8 and IL-18 Release and Disrupts TJ Proteins’ Gene Expression in LPS- and DSS-Stimulated Colonic Epithelial Cells

**Figure 7:**
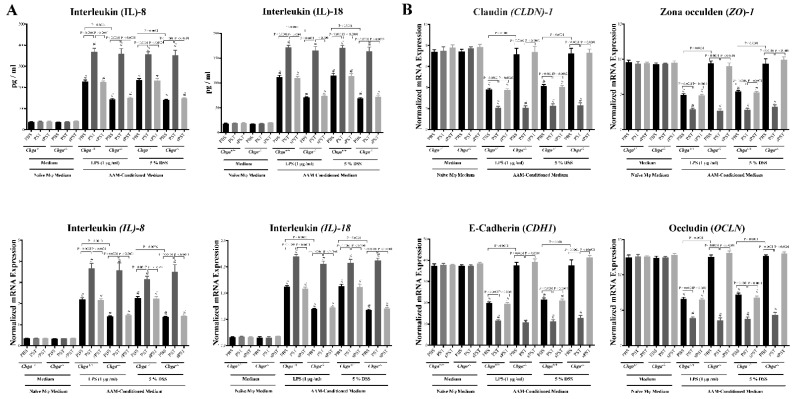
Conditioned medium isolated from pancreastatin (PST) pretreated naïve macrophages polarized toward an alternative-activated macrophages profile (AAM) increases IL-8 and IL-18 release and disrupts TJ proteins expression in Lipopolysaccharide (LPS)- and dextran sulfate sodium (DSS)-stimulated colonic epithelial cells. Peritoneal macrophages were isolated from naïve *Chga^+/+^* and *Chga^−/−^* mice. Macrophages were pretreated with PST or sPST (200 ng/mL) or 1% PBS for four hours, then stimulated by interleukin (IL)-4/IL-13 (20 ng/mL) for six hours and conditioned medium was harvested. Caco-2 cells were cultured in 2 mL supernatants of 1% phosphate buffer saline (PBS) or conditioned medium for 24 h, then challenged with LPS (1μg/mL) or 5% DSS for 24 h. Cells and supernatants were harvested for analysis. (**A**) Medium IL-8 and IL-18 protein levels and cell mRNA expression. (**B**) Cell mRNA expression of TJ protein (Claudin [*CLDN1*], zonula occludens-1 [*ZO1*], E-cadherin [*CDH1*], occludin [*OCLN*]). ELISA quantified protein level. Quantitative real-time RT–PCR quantified mRNA expression. The relative mRNA expression was determined by calculating the difference in the threshold cycle (ΔCt) method. One-way ANOVA followed by multiple comparison tests. Data represent mean ± SEM (*n* = 6). # refers to significance compared with control groups (naïve macrophage medium). Each experiment was repeated at least three times.

Next, we determined whether conditioned medium from PST-pretreated macrophages polarized to an AAM profile could regulate the release of IL-8 and IL-18 and gene expression of TJ proteins in human IECs following LPS or DSS-induced injury. Exposing Caco-2 cells to LPS (1 μg/mL) or 5% DSS for 24 h induced a significant increase of IL-8 and IL-18 release (Figure 7A) and a significant downregulation in mRNA expression of TJ proteins (*CLDN1*, *ZO1*, *CDH1*, *OCLN*) (Figure 7B). In both types of injury, conditioned medium of PST-pretreated *Chga^+/+^* macrophages polarized to an AAM significantly increased the release of IL-8 and IL-18 and significantly decreased the mRNA expression of TJ proteins when compared with conditioned medium of PBS-pretreated *Chga^+/+^* macrophages polarized to an AAM (Figure 7A,B). However, the conditioned medium of PBS-pretreated *Chga^−/−^* macrophages polarized to an AAM significantly decreased IL-8 and IL-18 and maintained mRNA expression of TJ proteins when compared with PBS-pretreated *Chga^+/+^* macrophages polarized to an AAM (Figure 7A,B). Finally, conditioned medium of PST-pretreated *Chga^−/−^* macrophages polarized to an AAM abolished these effects (Figure 7A,B). Pretreatment with sPST peptide did not show any significant impact on IECs function.

### 3.8. Conditioned Medium from PST-Pretreated Macrophages Polarized to an AAM Profile Delays the Intestinal Repair

**Figure 8:**
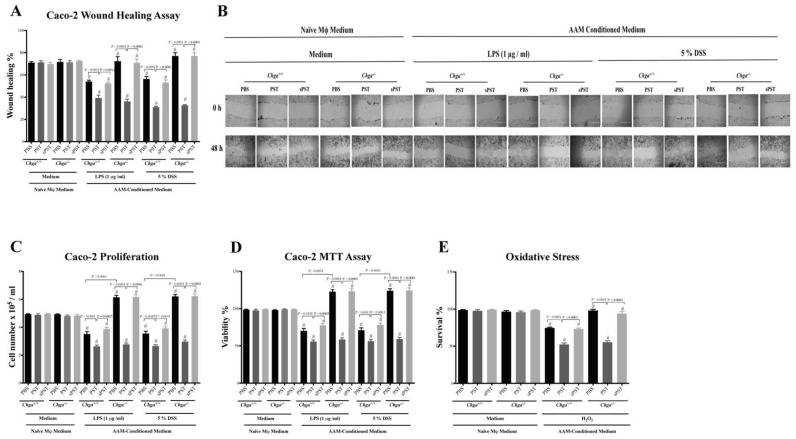
Conditioned medium isolated from pancreastatin (PST) pretreated naïve macrophages delays intestinal epithelial homeostasis. Peritoneal macrophages were isolated from naïve *Chga^+/+^* and *Chga^−/−^* mice. Macrophages were pretreated with PST or sPST (200 ng/mL) or 1% PBS for four hours, then stimulated by interleukin (IL)-4/IL-13 (20 ng/mL) for six hours and conditioned medium was harvested. Caco-2 cells were cultured in 2 mL supernatants of 1% phosphate buffer saline (PBS) or conditioned medium for 24 h, then challenged with LPS (1μg/mL) or 5% DSS for 24 h. (**A**,**B**) Epithelial cell migration assessed by wound healing assay (100×), (**C**) intestinal epithelial cell proliferation, (**D**) epithelial cell viability assessed by the 3-(4, 5-dimethyl thiazolyl-2yl)-2, 5-diphenyl tetrazolium (MTT) assay, and (**E**) epithelial cells oxidative stress assay from cultures treated with normal medium (control) or 200 mmol/L H_2_O_2_. One-way ANOVA was used to analyze the data, followed by multiple comparison tests. Data represent mean ± SEM (*n* = 6). # refers to significance compared to control groups. Each experiment was repeated at least three times.

AAM plays an essential role in intestinal homeostasis and epithelial repair during intestinal inflammation [4,9,13,18,25,41], and IBD involves functional impairment of IECs and infiltration of macrophages in the colonic mucosa [12,44]. Exposing Caco-2 cells to LPS (1μg/mL) or 5% DSS for 24 h induced a significant delay in the wound healing, proliferation, and viability of IECS (Figure 8A–D). Moreover, H_2_O_2_ caused a substantial reduction in cell survival compared with untreated cells (Figure 8E). In both types of injury, conditioned medium of PST-pretreated *Chga^+/+^* macrophages polarized to an AAM profile significantly delayed the wound healing, reduced proliferation and viability furthermore, and decreased the oxidative stress survivability of IECs when compared with conditioned medium of PBS-pretreated *Chga^+/+^* macrophages polarized to an AAM profile (Figure 8A–E). However, conditioned medium of PBS-pretreated *Chga^−/−^* macrophages polarized to an AAM profile significantly improved wound healing, proliferation and viability, and the oxidative stress survivability of IECs (Figure 8A–E). Finally, conditioned medium of PST-pretreated *Chga^−/−^* macrophages polarized to an AAM profile abolished these effects (Figure 8A–D). Pretreatment with sPST peptide did not show any significant impact on the functional capacity of IECs.

## 4. Discussion

This study tested a novel hypothesis that PST aggravates the development of colitis and worsens epithelial homeostasis. Herein, we showed that PST is increased in the colonic mucosa of participants with active UC, colitic mice and is associated with AAM altered functions and a disruption of the epithelial homeostasis. We demonstrated that PST exacerbated the disease onset and illness in DSS-induced colitis via downregulation of the anti-inflammatory and healing/repair processes. Furthermore, treatment with PST exacerbated the inflammation in DSS-treated mice by reducing the AAM activity and TJ proteins gene expression and enhancing the release of associated epithelial cytokines. We found that PST can indirectly exert its detrimental effects through additional mechanisms, including decreased epithelial proliferation, reduced epithelial wound healing, and deteriorated resistance to oxidative stress-induced apoptosis. This descriptive study collectively contributes new knowledge regarding PST’s detrimental actions and provides evidence for its proinflammatory role.

PST activity correlated with AAM and epithelial homeostasis functions in our five-models system: colonic biopsies from human subjects, colonic tissues from mice, ex vivo isolated macrophages from mice, in vitro polarized macrophages from mice, and in vitro human colonic epithelial cells line. This follows the data demonstrating that CHGA and its derived peptides play a significant role in macrophages activation during colitis progression [15,18,19,20,43]. In preclinical and clinical settings, CHGA level is increased and is correlated with the clinical severity of gastrointestinal inflammation in IBD patients [14,15] and in murine experimental models of colitis [15,20]. However, some contradictory data have demonstrated the protective effects of some of the CHGA-derived peptides against experimental colitis; the effect is associated with a decrease in proinflammatory macrophages and stimulation of AAM functions [18,19,20]. Conversely, the lack of CHGA in *Chga*-deficient mice demonstrated a reduction in the colitis severity associated with an increase in AAM’s polarization [15], implying the existence of a dominant proinflammatory CHGA-derived peptide. Our study showed that PST protein levels and mRNA activity increased within colonic biopsies from human participants with active UC compared with healthy controls. This is particularly convincing, given that EC produces CHGA and its derived peptides within the mucosa of the GIT and that a dramatically increased number of EC is seen in patients with active IBD [13,14]. In addition, we found that the PST level correlated negatively with AAM markers (IL-10, MR, CD1B) that are well-known to be altered during the development of IBD [5] and to play a critical role in epithelial repair [4,9].

In an experimental, translational plan, we demonstrated the existence of a deleterious proinflammatory role for PST during the development of experimental colitis. Here, PST worsened the disease activity and enhanced colonic damages associated with decreased AAM markers expression. This is an important feature, especially in the role portrayed by AAM in restraining the excessive inflammatory immune response and promoting tissue repair [5]. Several studies support our findings, demonstrating that mice with a deficient AAM polarization expressed higher susceptibility to colitis [6,45,46]. This is in accordance with the notion that polarized AAM drives a directionally concordant expansion of regulatory T cells to establish mucosal tolerance and protect against colitis [44]. Our markers analysis highlighting a downregulation of Ym1 and Arg1 is supported by data demonstrating that PST exerts a diabetogenic effect by increasing insulin resistance and promoting tissue inflammation by reducing AAM genes (*Ym1, Fizz1, Arg1*) [22]. In association with the results reported here, it is becoming increasingly possible that PST can exert a proinflammatory action within the intestinal mucosa.

Next, we assessed the impact of PST on collagen deposition. Colitis promotes collagen deposition, which alters the standard tissue architecture and results in fibrosis [32,47]. Our study showed that PST positively correlated with collagen expression in participants with active UC and that in mice, exogenous PST treatment enhanced significantly colonic collagen expression and deposition and in parallel to aggravating DSS-induced colitis. We speculate that PST alters the AAM-fibroblast interaction and increased collagen deposition and expression [48]. As demonstrated by other studies, it is also possible that AAM activation could decrease collagen deposition and protect against inflammation associated fibrosis [49,50]. In that context, a downregulation of AMM could increase collagen deposition and expression. Further studies are required to investigate PST’s effect on fibroblasts and AAM-Fibroblast interaction in the context of intestinal inflammation.

Several mechanisms have emerged and can contribute to the detrimental effects induced by PST. Therefore, we used LPS- and DSS-stimulated Caco-2 cells to investigate these mechanisms. LPS is an integral part of the intestinal inflammatory and is linked to the defective intestinal TJ barrier [51]. We demonstrated that polarized AAM pretreated with PST could disrupt epithelial homeostasis by increasing IL-8 and IL-18 release, reducing TJ proteins’ gene expression, and delaying wound healing processes in LPS- and DSS-Stimulated Caco-2 epithelial cells as well as decreasing the resistance of the epithelium to oxidative stress. This is in accordance with the alteration in epithelial homeostasis and TJ functions seen during intestinal inflammation development [52]. Our data are supported by evidence demonstrating protection against experimental colitis, minimizing mucosal damage and maintaining the epithelium equilibrium in IL-18 deficient mice [38,53]. Moreover, improving the epithelial cell’s functions, such as wound healing and resistance to oxidative, can result in the amelioration of colitis [54]. Therefore, this study is important for understanding PST’s effect on the cellular mechanisms that lead to intestinal tight junction barrier defects during intestinal inflammation and designing potential therapeutic strategies to promote retightening of the intestinal TJ barrier during IBD.

Surprisingly in our models, per se, PST does not affect basal conditions, implying that the peptides could only act in an inflammatory milieu.

Several limitations to our studies exist. First, although the mRNA levels do not correlate with protein expression levels or protein function [9], we quantified the mRNA levels based on the concept linking DNA, RNA and proteins and suggesting a direct relationship between mRNA and protein levels [25]. Second, in the absence of a cloned receptor, it would be essential to localize proteins’ expression and assess ex vivo intestinal permeability and colonic contractility permeability using USSING chamber and organ bath techniques. It is possible that other factors contributed to the effects of PST on intestinal inflammation in this study, especially the phosphorylation of phospholipase C activity and the activation of mitogen-activated protein kinases (MAPKs) and the GRP78 pathways [55]. Furthermore, gut microbiota plays a critical role in IBD pathophysiology, innate immunity, and epithelial homeostasis, and other CHGA-derived peptides have demonstrated an impact on the gut microbiota [23,56,57]. Therefore, further studies are necessary to explore the potential effects of PST on the activation of MAPK, permeability and gut microbiota during the progression of colitis.

## 5. Conclusions

Here, for the first time, we describe a deleterious effect of a proinflammatory peptide derived from CHGA, PST, in the context of colitis. These findings parallel our recent report demonstrating a beneficial impact of the deletion of *Chga* on colitis in *Chga^−/−^* mice [15]. This study confirmed that PST is elevated in human subjects with active UC and in an experimental colitis model. Moreover, we demonstrated the impact of a PST treatment on colitis using a murine model and its potential subsequent effects on AAM polarization and disruption of IECS homeostasis (see graphical abstract). This study provides a novel concept about PST as a potential biomarker to assess colitis’ clinical severity. Moreover, blocking or restraining PST activity in the intestinal mucosa could offer a new therapeutic window toward IBD.

## Figures and Tables

**Table 1:** Human primer sequences.

Gene Name	Forward	Reverse
*IL10*	GACTTTAAGGGTTACCTGGGTTG	TCACATGCGCCTTGATGTCTG
*MR*	GGAGTGATGGTTCTCCTGTTTC	CCTTTCAGCTCACCACAGTATT
*CD1B*	ACTCAGGAAATCCAATCCTCCTA	ATAGCAGGCTGTGAGCTACAT
*OCLDN*	ACAAGCGGTTTTATCCAGAGTC	GTCATCCACAGGCGAAGTTAAT
*TBP*	CCCGAAACGCCGAATATAATCC	AATCAGTGCCGTGGTTCGTG
*CLDN1*	AGGTGCTATCTGTTCAGTGATG	TGGCTGACTTTCCTTGTGTAG
*CADH1*	CTTCTGCTGATCCTGTCTGATG	TGCTGTGAAGGGAGATGTATTG
*ZO1*	CCAGCCTGCTAAACCTACTAAA	ATCTCTTGCTGCCAAACTATCT
*COL1A2*	GAGCGGTAACAAGGGTGAGC	CTTCCCCATTAGGGCCTCTC
*IL8*	ACTGAGAGTGATTGAGAGTGGAC	AACCCTCTGCACCCAGTTTTC
*IL18*	GCGTCACTACACTCAGCTAAT	GCGTCACTACACTCAGCTAAT
*CHGA Exon-VII*	GTTCCATGAAGCTCTCCTTCC	TCAAGGCTGTCCTCCCA

**Table 2:** Mouse primer sequences.

Gene	Forward	Reverse
*Il10*	GCTCTTACTGACTGGCATGAG	CGCAGCTCTAGGAGCATGTG
*Arg1*	TTGGGTGGATGCTCACACTG	GTACACGATGTCTTTGGCAGA
*Il18*	GACTCTTGCGTCAACTTCAAGG	CAGGCTGTCTTTTGTCAACGA
*Ym1*	CAGGTCTGGCAATTCTTCTGAA	GTCTTGCTCATGTGTGTAAGTGA
*Fizz1*	AAGCCTACACTGTGTTTCCTTTT	GCTTCCTTGATCCTTTGATCCAC
*Col1a2*	GGTGAGCCTGGTCAAACGG	ACTGTGTCCTTTCACGCCTTT
*Eef2*	TGTCAGTCATCGCCCATGTG	CATCCTTGCGAGTGTCAGTGA
*Ocldn*	TTGAAAGTCCACCTCCTTACAGA	CCGGATAAAAAGAGTACGCTGG
*Cldn1*	GGGGACAACATCGTGACCG	AGGAGTCGAAGACTTTGCACT
*Zo1*	GCCGCTAAGAGCACAGCAA	TCCCCACTCTGAAAATGAGGA
*Cadh1*	CATCCCAGAACCTCGAAACA	TGGGTTAGCTCAGCAGTAAAG

## Supplementary Materials

The following are available online at https://www.mdpi.com/2227-9059/9/2/134/s1.
