# Supplementary material for: Pancreastatin Reduces Alternatively Activated Macrophages, Disrupts the Epithelial Homeostasis and Aggravates Colonic Inflammation. A Descriptive Analysis
Source: Biomedicines. 2021 Feb 1;9(2):134. doi: 10.3390/biomedicines9020134 (PMC7912769; doi:10.3390/biomedicines9020134)
Supplement: Supplementary file 1 [file biomedicines-09-00134-s001.pdf]

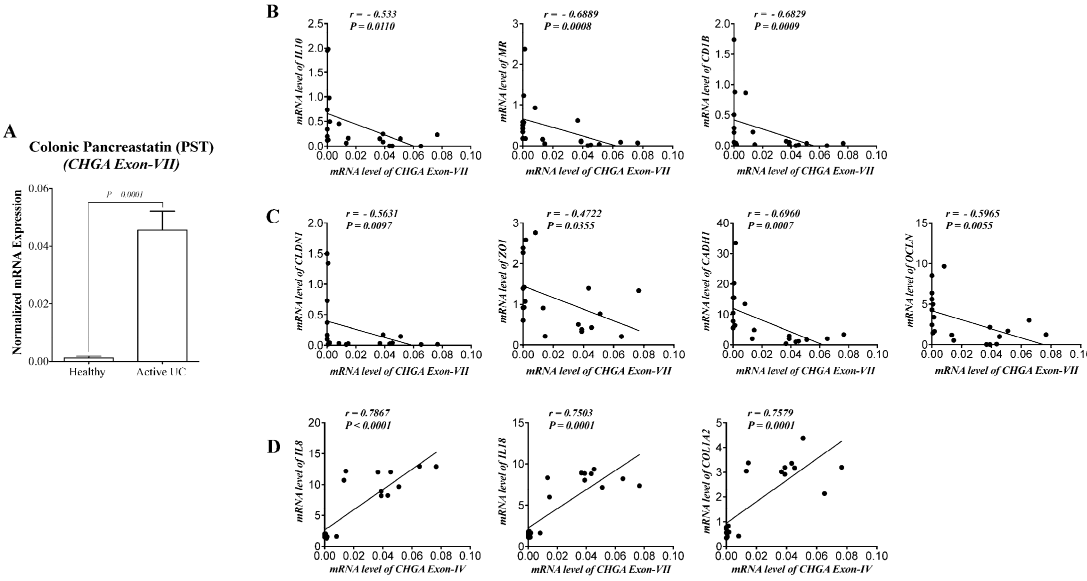

Figure S1. Chromogranin-A Exxon VII (Pancreastatin (PST)) expression is reduced in participants with active ulcerative colitis (UC) and correlates negatively with mRNA alternative-activated macrophages (AAM) markers and tight junction (TJ) protein, and correlates positively with epithelial cells-associated cytokines and collagen expression. *(A)* Chromogranin-A Exxon VII mRNA expression in UC participants' colonic tissue (n=10) and healthy individuals (n=10). Correlation analysis between Chromogranin-A exxon VII mRNA expression and mRNA levels of *(B)* AAM markers (*IL10,* Mannose receptor *[MR],* Cluster of differentiation 1B *[CD1B]*), *(C)* TJ proteins (Claudin *[CLDN1],* zonula occludens-1 *[ZO1],* E-cadherin *[CDH1]* and occludin *[OCLN]*), *(D)* and *IL8*, *IL18*, and collagen (*COL1A2*) in human. mRNA expression was quantified by quantitative real-time RT-PCR. Mann-Whitney test and Spearman's correlation were used to analyze the data. Two tails significance level adjusted at 0.05.
